# Assessment of COVID-19 vaccines acceptance in the Lebanese population: a national cross-sectional study

**DOI:** 10.1186/s40545-021-00403-x

**Published:** 2022-01-11

**Authors:** Philippe Hanna, Aline Issa, Ziad Noujeim, Mira Hleyhel, Nadine Saleh

**Affiliations:** 1 Faculty of Public Health, Lebanese University, Fanar, Lebanon; 2 Department of Oral and Maxillofacial Surgery, Faculty of Dental Medicine, Lebanese University, Hadath, Lebanon; 3 Department of Oral Medicine and Maxillofacial Radiology, Faculty of Dental Medicine, Lebanese University, Hadath, Lebanon; 4 INSPECT-LB (Institut National de Santé Publique, Epidémiologie Clinique et Toxicologie-Liban), Beirut, Lebanon

**Keywords:** COVID-19, Vaccines, Acceptance, Middle East, Lebanon

## Abstract

**Background:**

Vaccines have become the best weapon for epidemic prevention and control in the absence of standard approved effective therapies. However, skepticism about the vaccine efficacy and safety is constantly reported. To our knowledge, there has been no study assessing COVID-19 vaccine acceptance in Lebanon. The primary objective of this survey is to assess the COVID-19 vaccines’ acceptance and its related determinants in the Lebanese population.

**Methods:**

A cross-sectional study was conducted in Lebanon from February 16 through February 25, 2021. Data was collected using an online questionnaire via social media platforms using the snowball technique. The questionnaire consisted of 47 questions related to sociodemographic and medical history, COVID-19 experience, knowledge, practice, and beliefs towards COVID-19 vaccines, including vaccines acceptance. Binary logistic regression was performed to identify factors associated with vaccine acceptance.

**Results:**

A total of 1209 questionnaires were completed; around 63.4% have reported their acceptance for receiving the COVID-19 vaccine, while only 57% of participants registered themselves on the national platform. The multivariable analysis showed that a higher knowledge scale, living in an urban residential area, having hypertension, not having a food allergy, reporting a higher fear to experience COVID-19 infection, and receiving or wanting to receive influenza vaccine, were positive predictors of COVID-19 vaccines acceptance.

**Conclusions:**

Our findings support the need to improve knowledge about COVID-19 infection and vaccination through education and awareness programs. Specifically residents of rural areas should be targeted to optimize COVID-19 vaccine acceptance among the Lebanese population.

**Supplementary Information:**

The online version contains supplementary material available at 10.1186/s40545-021-00403-x.

## Background

The emergence of the severe acute respiratory syndrome coronavirus-2 (SARS-CoV-2) virus, also known as COVID-19 has prompted the World Health Organization (WHO) to declare this disease as a public health emergency in late January 2020 [1] and characterized the novel virus as a pandemic in March 2020 [2]. As of September 11, 2021 over 224 million confirmed cases have been diagnosed globally with more than 6.63 million deaths [3]. Patients can experience a range of clinical manifestations, from no symptoms to critical illness [3]. The prognosis of the elderly and those with chronic underlying systemic diseases is poor [2]. Based on recent reports, around 597,000 new cases are being reported daily, and the total active hospitalized cases worldwide reported as serious and critical accounts for around 103,000 [3]. The symptoms of infected children are relatively mild [3].

In Lebanon, a total of 610,197 COVID-19 infections have been reported as of September 2021, with 8144 deaths [4]. Yet it is essential to highlight that at the time of the study, the country was facing wave 3 of the pandemic with around 4000 new cases daily that are draining the healthcare professionals and oversaturating the hospitals’ capacity [4]. The consequences of the COVID-19 pandemic have been amplified in Lebanon due to the Beirut Blast on August 4, 2020 [5] and the economic crisis that the country is still facing due to Lebanese Pound devaluation, which further amplified the scarcity of medications [6], the medical devices, and supplies [6].

As of June 2021, there are no approved medications for the treatment of COVID-19 [7]. Management of the disease is based on supportive care and emerging medications with potential protective effects. Current options include the antiviral remdesivir and the anti-inflammatory steroid dexamethasone in adult patients with severe disease [7]. Yet, there is no consensus for standard management guidelines for the pandemic [7]. Several measures have been sequentially implemented to minimize the infection rate, including enforcement of quarantine and lockdowns, social distancing, community use of face masks at all times, and travel restrictions [6, 8]. Therefore, vaccines have become the best weapon for epidemic prevention and control [9].

According to public reports, more than 118 companies and scientific research institutions worldwide are developing 214 COVID-19 vaccine projects [9]. As of June 2021, a total of 13 vaccines have been approved by at least one country including two mRNA vaccines (Pfizer/BioNtech and Moderna), three Vector vaccines (Johnson and Johnson, Sputnik V, and AstraZeniza/Oxford) in addition to inactivated virus vaccines (Sinofarm & Sinovac) [10].

However, discussions related to the rapid development of COVID-19 vaccines, or their safety have been the reasons for debates over the acceptance or rejection of the vaccine’s products by the public [11]. These discussions include skepticism on the vaccine’s efficacy or even conspiracy theories about the emergence of the virus [11]. In fact, literature have reported a range of vaccination acceptance rates that varied over time and across countries from 43 to 93% [12, 13].

In Lebanon, the national vaccination campaign against COVID-19 was launched on February 14, 2021 [14]. However, there has been no data related to COVID-19 vaccines acceptance. Yet, data from neighboring countries have noted a sharp decrease in hospitalization with advanced vaccination rate [15]. Therefore, the main objective of this survey was to assess the COVID-19 vaccines’ acceptance and its related determinants in the Lebanese population.

## Methods

### Study design

This study was an observational cross-sectional study conducted in Lebanon from February 16 to February 25, 2021, using an online survey.

### Study population and sample size

Participants were eligible to be included in the study if they were adults (aged 18 years or older) and residing in Lebanon at the time of the survey. Participants who were below 18 years of age or not residing in Lebanon as well as those who did not give their consent to participate were excluded from the study.

The sample size was calculated using Epi Info 7 StatCalc functions for a population survey. Since the prevalence rate of COVID-19 vaccine acceptance in some neighboring Arab countries [12, 16–18] varied between 30.9% % and 60.7%, we assumed that the expected acceptance prevalence in Lebanon would be around 50%. Based on the 50% expected prevalence, the minimal sample size calculated was 384 with a confidence level of 95% and a margin of error of 5%.

### Survey development

A group of health professionals with extended experience in public health and academia developed the questionnaire based on a thorough review of the literature [11–13, 16–31] and guidelines issued by health organizations [30, 31]. The questionnaire was piloted and reviewed by a set of 5 other health professionals. All questions were simplified for better understanding (Additional file 1: Appendix 1: Questionnaire - English Version). The final questionnaire required between 10 and 15 min to complete. The questionnaire was developed in two languages, English and Arabic to maximize comprehension and public participation. The tool consisted of 47 questions using a combination of Likert scales and multiple-choice questions. It consisted of the following sections: sociodemographic characteristics, experience with COVID-19, knowledge, practice, and beliefs.

### Procedure

The study was performed at the time when a lockdown was implemented in Lebanon. Therefore, a snowball sampling method was used using online Google forms (https://forms.gle/fTTsePe9sz9cq82p7) [32]. The survey was distributed through social media platforms, including WhatsApp and Facebook.

### Ethical considerations

Being observational, voluntary, and respecting participants’ anonymity and confidentiality, the Institutional Review Board (IRB) of the Lebanese University in Beirut—Lebanon; waived the need for ethical approval. Data were collected anonymously with no identifying or sensitive information.

### Statistical analysis

Descriptive statistics were performed using means and standard deviations for quantitative continuous variables and frequencies and percentages for categorical variables. Knowledge related to COVID-19 vaccines was assessed with 15 questions. Each question was given a weight of one point. Every correct answer added on the knowledge index one point, whereas a wrong or an “I don’t know” answer was given zero (0) point. The total knowledge index ranged from 0 to 15 with a higher score indicating better knowledge. A modified Bloom’s cutoff point (15) was used to categorize the index into three levels of knowledge: high (80–100% score range 12–15) moderate (50–79% score range 8–11), and low (0–50% score range 0–7).

Binary logistic regression analysis was carried out using the acceptance of COVID19 vaccination as the dependent variable. Independent predictors associated with the dependent variable with a *p* value of 0.2 or less in the bivariate analysis were included in the multivariable model. Adjusted odds ratio (aOR) and their 95% intervals (95% CI) were calculated. Statistical analyses were performed using the Statistical Package for the Social Sciences for Windows, Version 23.0 (IBM SPSS Statistics, Armonk, NY, USA).

## Results

### Patients’ socio-demographics characteristics and medical history

Out of 1279 participants who responded to the survey link, 70 participants were excluded for the following reasons: Did not wish to participate (7), younger than 18 years (15), and living outside Lebanon (48). The final sample included a total of 1209 participants who filled out the survey. Participants’ characteristics are addressed in Table 1. The majority of the participants were Lebanese (98.6%) aged between 18 and 80 years. The average body mass index of the sample was 25.12 ± 4.89 (kg/m^2^). Most of the participants have achieved a university level, with 39% having post-graduate education. The most frequently reported comorbidities were obesity (14%), followed by hypertension (10.3%), respiratory diseases (5%), and diabetes (3.8%). Only 26.6% reported living with the elderly and 25.2% with individuals with chronic diseases. Around 12% of the participants reported having allergies to medications. Among 249 participants who reported having ever been infected with COVID-19, only 7.5% experienced severe symptoms. The majority of participants reported knowing a family member or friend who had a severe COVID-19 infection. Participants reported an average of 6.35 over 10 for the fear of getting the COVID-19 infection scale.

**Table 1:** Sociodemographic characteristics and medical history of study participants (*N* = 1209)

Participants characteristic	Frequency (percentage)
Age (years)	38.86 ± 12.47
Gender	
Males	398 (32.9)
Females	811 (67.1)
Nationality	
Lebanese	1193 (98.67)
Syrian	10 (0.82)
Palestinian	4 (0.33)
Other	28 (2.31)
Pregnant	11/811(1.4)
Breastfeeding	26/811(3.2)
Governorates	
Beirut	257 (21.3)
Mount Lebanon	765 (63.3)
Northern Lebanon	77 (6.4)
Southern Lebanon	74 (6.1)
Bekaa	36 (3.0)
Residence area	
Urban	896 (74.1)
Rural	313 (25.9)
Marital status	
Single	500 (41.4)
Married	655 (54.2)
Other^a^	54 (4.4)
Living in the same household	
With children	542 (44.8)
With elderly	322 (26.6)
Individuals with chronic diseases	305 (25.2)
Educational level	
School	101 (8.4)
University	638 (52.8)
Advanced degree	470 (38.9)
Employment status	
Employed	904 (74.7)
General risk job	379 (31.3)
Moderate risk job	335 (27.7)
High-risk job	190 (15.7)
Unemployed + retired	305 (25.3)
Comorbidities	247 (20.4)
Diabetes	46 (3.8)
Hypertension	125 (10.3)
Cardiac conditions	26 (2.2)
Malignancies	8 (0.7)
Kidney and liver diseases	11 (0.9)
Thromboembolic diseases	7 (0.6)
Respiratory diseases	60 (5)
Auto-immune diseases	28 (2.3)
Body Mass Index (BMI)	
Underweight	52 (4.3)
Normal weight	606 (50.4)
Overweight	373 (31.0)
Obese	171 (14.2)
Allergies	
To medications	141 (11.7)
To food	160 (13.2)
Social history	
Smoking	385 (31.8)
Previous COVID-19 infection	
No	960 (79.4)
Yes, with mild symptoms	109 (9.0)
Yes, with moderate symptoms	114 (9.4)
Yes, with severe symptoms	18 (1.2)
Family or friend who had severe COVID-19	925 (76.5)
Fear Index (over 10)	6.35 ± 3.1
Attitude toward medications	
Don’t like to take	174 (14.4)
Only when prescribed	595 (49.2)
Take when needed	440 (36.4)
Taken or willing to take Influenza vaccine	576 (47.6)
Attitude toward vaccination in general	
In favor of vaccination	1017(84.1)
Against vaccination	102 (8.4)
Hesitant towards vaccination	90 (7.4)

^a^Other (Divorced and widowed)

Around half of participants (49.2%) reported taking medication only when prescribed, while 47.2% reported having received influenza vaccine previously or planning to receive it in the future. Around 84% of respondents expressed a favorable attitude towards vaccination in general.

### Knowledge and sources of information about COVID-19 vaccines

The mean knowledge scale towards COVID-19 vaccines was 10.51 ± 2.93, with Cronbach alpha for internal consistency estimated at 0.79. While 32.3% of the participants achieved a high COVID-19 vaccines’ knowledge level, 42.1% and 25.7% achieved intermediate and low knowledge levels, respectively. Participants’ responses to each of the 15 knowledge questions are presented in Table 2. Regarding the reported sources of information about COVID-19 vaccines, the majority of respondents reported consulting with scientists and scientific releases (60.5%), about half reported consulting the Ministry of Public Health website (55.3%), World Health Organization website (52%), and pharmacists (47.6%), and only 18.6% reported consulting primary care physicians. Participants also reported television (54.4%), social media platforms (30.7%), and friends or family members (17.9%) as sources of information about COVID-19 vaccines.

**Table 2:** Responses to questions about COVID-19 vaccines knowledge by the study participants

	Correct answer	*n* (%)
1. COVID-19 vaccines decrease the risk of symptomatic infection with the COVID-19 virus	True	965 (79.8)
2. COVID-19 vaccines decrease the risk of transmission of the COVID-19 virus	True	679 (56.2)
3. All available vaccines produce antibodies against COVID-19	True	685 (56.7)
4. COVID-19 vaccines provide you with immediate protection directly after the first dose	False	810 (67.0)
5. Johnson and Johnson’s vaccine is given in two doses	False	356 (29.4)
6. Al COVID-19 vaccines preparation techniques are new and were never used before	False	649 (53.7)
7. COVID-19 vaccine is an effective treatment of active COVID-19 infection	False	532 (44.2)
8. Most of the confirmed side effects of the COVID-19 vaccines are mild, resolving in 2–3 days	True	678 (56.1)
9. People who were previously infected with COVID-19 will need to be vaccinated for COVID-19 at a certain time	True	995 (82.3)
10. The faster the people will become vaccinated, the probability of the appearance of new variants of the virus will decrease (UK variant, South African, etc.…)	True	857 (70.9)
11. It is preferable that the two doses of the vaccine given to an individual be from the same brand	True	923 (76.3)
12. Vaccinated people will not need to take preventive measures	False	932 (77.1)
13. Anyone can take the COVID-19 vaccine	False	662 (54.8)*
14. Influenza vaccine protects against COVID-19	False	913 (75.5)
15. COVID-19 vaccines contain microchips influencing our body and brain	False	827 (68.4)

(*) Not statistically significant result (*p* ≥ 0.05)

### Acceptance of COVID-19 vaccines, motivators, and barriers

More than half of the participants (63.4%) reported their acceptance to receive COVID-19 vaccines. Regarding the registration in the Lebanese Ministry of Public Health platform to receive the vaccine, 57% of participants reported being registered, while 66% reported having registered their family members.

**Fig. 1:**
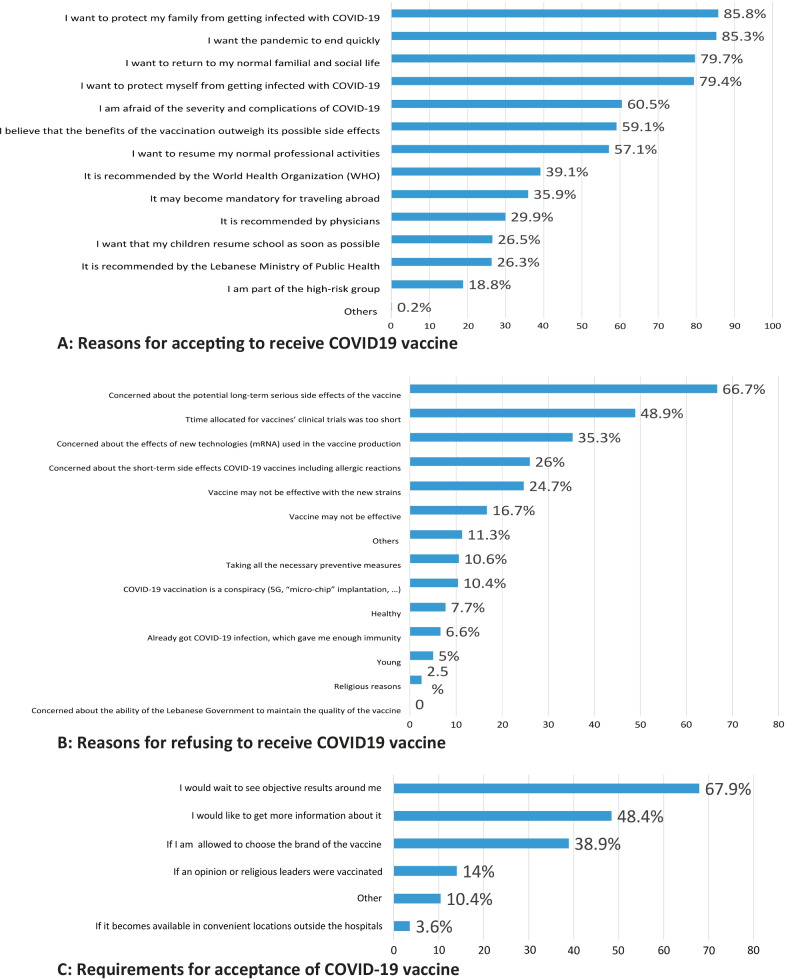
**A** Reasons for accepting to receive COVID19 vaccine. **B** Reasons for refusing to receive COVID19 vaccine. **C** Requirements for acceptance of COVID-19 vaccine

The most common reasons given by participants for accepting to receive the vaccine were protecting their family members from getting infected with COVID-19 (85.8%), wishing to end the pandemic quickly (85.3%), and wishing to return to normal life with family and friends (79.7%), and protecting themselves from getting infected with COVID-19 (79.4%) (Fig. 1A).

The most common barriers for refusing to receive the vaccines included participants’ concern about the vaccines’ potential long-term serious side effects (66.7%), the short time allocated to vaccines’ clinical trials (48.9%), the effects of new technologies (mRNA) used in the vaccine production (35.3%), and short-term side effects of the vaccines including allergic reactions (26%) (Fig. 1B). Participants who reported refusal to receive the vaccines highlighted that they need to observe positive vaccines results in people they know (68%), to receive more information about COVID-19 vaccines (48.4%), and to be able to select the vaccine brand to receive (38.9%) to change their mind about refusing COVID-19 vaccine (Fig. 1C).

### Determinants of COVID-19 vaccines acceptance

In the multivariable analysis (Table 3), several variables were positively associated with higher vaccine acceptance. A higher knowledge index (aOR = 1.271; CI 1.218–1.326), reporting hypertension as comorbidity (aOR = 1.698; CI 1.042–2.767). Furthermore, participants who reported a higher fear scale (aOR = 1.057; CI 1.012–1.104), and who indicated receiving influenza vaccines (aOR = 3.123; CI 2.369–4.117) showed a higher COVID-19 vaccine acceptance. On the other hand, living in Rural area (aOR = 0.703; CI 0.504–0.908), having reported food allergy (aOR = 0.644; CI 0.433–0.958) showed higher COVID-19 vaccine hesitancy.

**Table 3:** Multivariable logistic regression model for the determinants of patient’s acceptance to COVID-19 vaccines

Variable	ORa	Confidence interval	*p* value
Governorates			0.053
Beirut	1		
Mount Lebanon	0.741	0.520–1.057	0.098
Northern Lebanon	0.561	0.303–1.042	0.067
Southern Lebanon	1.493	0.741–3.008	0.262
Bekaa	1.032	0.444–2.398	0.943
Area			
Urban	1		
Rural	0.703	0.504–0.980	0.038
Body Mass Index			0.094
Underweight	1		
Normal weight	1.021	0.521–2.000	0.952
Overweight	1.498	0.746–3.007	0.256
Obese	1.011	0.477–2.140	0.978
Hypertension			
No	1		
Yes	1.698	1.042–2.767	0.034
Food allergy			
No	1		
Yes	0.644	0.433–0.958	0.030
Influenza vaccine			
No	1		
Yes	3.123	2.369–4.117	< 0.001
Fear Scale	1.057	1.012–1.104	0.013
Knowledge Scale	1.271	1.218–1.326	< 0.001

Variables with a *p* value of 0.2 or less in the bivariate analysis were included in the initial model. Variables included in the multivariable logistic regression model: gender, marital status, governorates, education, occupation, knowledge index, urban/rural, living with an elderly, smoking, hypertension, food allergy, fear scale, friend/family having COVID-19, medication in general, body mass index, age, influenza vaccine. Using the Backward Stepwise regression Model, the model finally retained the variables shown in this table. Hosmer and Lemeshow test for sample adequacy *p* value: 0.84

## Discussion

To our knowledge, this is the first study that evaluated the acceptance of the COVID-19 vaccine and its determinants in the Lebanese population. Our results showed that the vaccine acceptance was rated as moderate to high among the survey respondents. Our results show that high vaccine acceptance is predicted by having a high knowledge regarding the vaccine, having hypertension, also fear from the COVID-19 infection is a predictor of high acceptance level. Being vaccinated or having a positive attitude towards the influenza vaccine plays a role as a predictor of high vaccine acceptance. On the other side, low vaccine acceptance is predicted by living in rural areas and having a food allergy.

The acceptance rate in our study was similar to that reported in KSA [17] (64.7%) and several western countries [21, 23, 25, 26] including the US (69%) [21], Japan (65.7%) [23] and the UK (64–71.7%) [25, 26] compared to a lower rate reported in Kuwait (53.1%) [18]. It may be argued that the results may be an overestimation of the acceptance to be vaccinated, since the respondents are predominantly highly educated individuals. Sequentially the sample is not representative of the normal mix of the population. However, this overestimation can also be counteracted and even attenuated by the fact that our population is younger than the average population, majority females (67.1%) and having fewer comorbidities (20.4%) than the public. Hence, less implicated with COVID-19 risk factors, and less fear from the disease [2–4]. Furthermore, at the time of the study, the public was not fully cognizant of the risks of the disease nor the value of the vaccines. Moreover, the vaccine was not popular nor indicated for young people due to vaccine priority and national plans.

It is postulated that the acceptance rate is highly influenced by the timing of the study. In that perspective, our study was launched at the beginning of the national vaccination campaign, where there have been several official releases as well as a media campaign, this may have contributed to having an appropriate level of knowledge toward COVID-19 vaccines among respondents in Lebanon (10.51 ± 2.93; 32.3% high knowledge level). These data contrast with studies [33] conducted earlier during the pandemic, where an alarming low vaccine acceptance rate (26%) was reported among adults in Europe across seven European countries, including the UK [33]. Participants were unsure or unwilling to get a COVID-19 vaccine when available. Other studies have found that around one-quarter of French and US adults do not intend to receive the vaccine even if offered for free [33].

Our study participants had in general an acceptable level of knowledge toward COVID-19 vaccines: 74.4% answered correctly more than 50% of the knowledge questions.; participants were able to answer correctly questions related to vaccines’ effect, doses, expected adverse drug events, immunity timeframe after vaccination, as well as protective measures post-vaccination. Such results were expected, considering that the study was conducted in mid-February, with the beginning of the national vaccination campaign of the Lebanese Ministry of Public Health, and after several official information regarding the value of the COVID-19 vaccination, communicated to the general public through mass media [14]. In addition, these results can be further explained by the demographic characteristics of our sample that included young, highly educated respondents who are known to be more familiar with technology and consequently have better access to social media platforms, compared to elderly participants. The knowledge level in our study was higher than that reported in a recent study in Bangladesh which used the same methodology and showed an overall rate of correct vaccine knowledge answers of 57% [34]. However, our study reported a lower percentage of knowledge for questions that has been less addressed in media, such as the number of doses for the Johnson and Johnson’s vaccine. At the time of the survey, the latter vaccine was still not approved by the FDA and not yet available in Lebanon and sequentially it was not discussed as much as the other vaccines [14].

Regarding the source of information, our results revealed that participants sought information on COVID-19 vaccines from various sources including scientists and scientific releases, the Lebanese MoPH website, the World Health Organization, pharmacists, and primary care physicians. Thus, respondents were using credible information sources. However, a significant number still relied on television, social platforms, and even friends and family members to get information.

Similar rates for using trusted and unreliable sources of COVID-19 information have been previously reported in Lebanon [6] Similarly, other studies published in Middle Eastern countries, particularly Jordan and Kuwait reported getting information about COVID-19 vaccines from television (31.7%), social media platforms (30.1%), compared to only 36.4% from trusted sources [16], this can lead to a high level of misinformation and sequentially may increase hesitancy toward vaccinations [16].

Multivariable analysis showed that living in the Northern Lebanon governorate is more likely to decrease the vaccine acceptability compared to those living in Beirut. Furthermore, living in rural areas reduces the likelihood of accepting the COVID-19 vaccines by 30%. Similarly, a study conducted in the United States in October 2020, showed a lower rate of acceptance in rural areas, this result was linked to a lower knowledge regarding COVID-19 vaccines [35]. In fact, studies [36, 37] have reported that individuals living in rural areas have higher fear of COVID-19 vaccines, and sequentially, the fear of adverse effects frequency is increasing the vaccination hesitancy.

Our findings also showed that patients with hypertension are more likely to accept the vaccines compared to those without hypertension. Hypertension is a chronic and comorbid condition and sequentially, due to its high prevalence, it is well represented in our sample. A study [38] assessing COVID-19 vaccine hesitancy in northern Italy highlighted that the lack of comorbidities is a predictor of hesitancy. On another note, people may have hesitated to take the vaccines because of fear of allergies. No evidence suggests that food allergies may predispose to higher degree of allergies. Therefore, the effect of food allergies on vaccines’ acceptance may be an incidental finding.

While there has been some relationship among those who accept COVID-19 vaccines and influenza vaccine, only 47% of our respondents have either received or willing to receive the influenza vaccines, while a total of 63.4% indicated that they accept COVID-19 vaccines.

Similarly, a nationwide study conducted on US adults and published in February 2021 demonstrated that having risk factors increased the likelihood to be vaccinated and highlighted the positive impact of influenza vaccine acceptance on vaccine acceptance which aligns with our study results [20]. Previous studies highlighted that a history of vaccination against seasonal influenza was an independent predictive factor for the acceptance of COVID-19 vaccines [13, 27]. In addition, most of those who reported acceptance toward COVID-19 vaccines registered themselves on the national platform, but only 66.3% registered their family members.

Interestingly, our study results highlighted that the higher the COVID-19 fear scale, the higher the participant’s willingness to take the vaccines; this finding aligns with literature [17, 18, 21, 39] that reported that the willingness to take the vaccines is higher when the risk of getting the disease is high. The fear of being infected with COVID-19 is identified as a predictive factor for COVID-19 acceptance; it increased the acceptance by 2.13 times among survey participants in the KSA. Along the same lines, our results indicated that the knowledge index is a positive predictor of COVID-19 acceptance, such as studies in the USA [20, 27], UK [26], and Greece [28].

## Strengths and limitations

To the best of our knowledge, this is the first study in Lebanon that assessed the acceptance of COVID-19 vaccines and their determinants in the Lebanese population. Moreover, the online questionnaire led to wide accessibility among the participants all over Lebanon; despite having lower representability from the Bekaa area, which is a distant area from the capital, suggesting a probable overestimation of the obtained results. Furthermore, using the snowball technique may be another limitation. The results of our analysis cannot be generalized to the entire Lebanese Community, since our sample consisted of more females, educated and young public, with easy access to social media; A selection bias may have existed as the methods may have discriminated against older adults or those with poor internet access or with lower computer literacy. Finally, the questionnaire is self-administered which suggests the possibility of having information bias. However, piloting the questionnaire and revising it for clarity and content before releasing the final form, should have minimized the risk of misunderstanding the questions.

## Conclusion

This cross-sectional study revealed a high acceptance of COVID-19 vaccines among the Lebanese population. Our findings revealed a significant predictor of the COVID1-19 vaccine to be general vaccine knowledge index, fear index, and acceptance to receive influenza vaccine. Officials and stakeholders should tackle factors associated with vaccine acceptance to increase the vaccination rate and achieve herd immunity. Targeted educational campaigns, especially in rural areas, should be prioritized by municipalities and media to maximize awareness and increase the acceptance of COVID-19 vaccines.

## Supplementary Information

**Additional file 1: Appendix 1**. Questionnaire—English Version.

## Data Availability

The data sets used and/or analyzed during the current study are available from the corresponding author on reasonable request. All data generated or analyzed during this study are included in this published article.

## References

[1] COVID-19 pandemic remains public health emergency of international concern. 2020. https://www.euro.who.int/en/health-topics/health-emergencies/coronavirus-covid-19/news/news/2020/5/covid-19-pandemic-remains-public-health-emergency-of-international-concern. Accessed 9 June 2021.

[2] Coronavirus disease (COVID-19)—World Health Organization. Who.int. https://www.who.int/emergencies/diseases/novel-coronavirus-2019. Accessed 9 June 2021.

[3] COVID live update: 166,287,417 cases and 3,452,766 deaths from the Coronavirus—worldometer. Worldometers.info. https://www.worldometers.info/coronavirus/. Accessed 9 June 2021.

[4] COVID-19 Coronavirus Lebanon Cases.gov.lb. http://www.moph.gov.lb/maps/covid19.php. Accessed 9 June 2021.

[5] Bostock B. Lebanon’s devastating blast came in the middle of an unprecedented economic crisis, frequent power outages, and hospitals struggling to contain the coronavirus. Business Insider. 2020. https://www.businessinsider.com/beirut-explosion-lebanon-buckling-economic-crisis-outages-coronavirus-2020-8. Accessed 9 June 2021.

[6] Zeenny RM, Ramia E, Akiki Y, Hallit S, Salameh P (2020). Assessing knowledge, attitude, practice, and preparedness of hospital pharmacists in Lebanon towards COVID-19 pandemic: a cross-sectional study. J Pharm Policy Pract.

[7] Sanders JM, Monogue ML, Jodlowski TZ, Cutrell JB (2020). Pharmacologic treatments for coronavirus disease 2019 (COVID-19): a review. JAMA.

[8] Replication-receiver. COVID-19 pandemic. UNDP. 2020. https://www.latinamerica.undp.org/content/rblac/en/home/coronavirus.html. Accessed 9 June 2021.

[9] Draft landscape and tracker of COVID-19 candidate vaccines. Who.int. https://www.who.int/publications/m/item/draft-landscape-of-covid-19-candidate-vaccines. Accessed 9 June 2021.

[10] Vaccines—COVID19 Vaccine Tracker. Trackvaccines.org. https://covid19.trackvaccines.org/vaccines/. Accessed 9 June 2021.

[11] COVID-19 Mythbusters—World Health Organization. Who.int. https://www.who.int/emergencies/diseases/novel-coronavirus-2019/advice-for-public/myth-busters. Accessed 9 June 2021.

[12] Mannan AK, Farhana KM. Knowledge, attitude and acceptance of a COVID-19 vaccine: a global cross-sectional study. Munich Personal RePEc Archive; 2020. https://mpra.ub.uni-muenchen.de/105236/. Accessed 11 Sept 2021.

[13] Verger P, Scronias D, Dauby N, Adedzi KA, Gobert C, Bergeat M (2021). Attitudes of healthcare workers towards COVID-19 vaccination: a survey in France and French-speaking parts of Belgium and Canada, 2020. Euro Surveill.

[14] COVID-19 Vaccine. Gov.lb. https://www.moph.gov.lb/en/covid-vaccine. Accessed 9 June 2021.

[15] Radcliffe S. COVID-19 cases dropping in groups with high vaccination rate. Healthline Media. 2021. https://www.healthline.com/health-news/covid-19-cases-dropping-in-groups-with-high-vaccination-rate. Accessed 9 June 2021.

[16] Sallam M, Dababseh D, Eid H, Al-Mahzoum K, Al-Haidar A, Taim D (2021). High rates of COVID-19 vaccine hesitancy and its association with conspiracy beliefs: a study in Jordan and Kuwait among other Arab countries. Vaccines (Basel).

[17] Al-Mohaithef M, Padhi BK (2020). Determinants of COVID-19 vaccine acceptance in Saudi Arabia: a web-based national survey. J Multidiscip Healthc.

[18] Alqudeimat Y, Alenezi D, AlHajri B, Alfouzan H, Almokhaizeem Z, Altamimi S (2021). Acceptance of a COVID-19 vaccine and its related determinants among the general adult population in Kuwait. Med Princ Pract.

[19] Pastorino R, Villani L, Mariani M, Ricciardi W, Graffigna G, Boccia S (2021). Impact of COVID-19 pandemic on flu and COVID-19 vaccination intentions among university students. Vaccines (Basel).

[20] Ruiz JB, Bell RA (2021). Predictors of intention to vaccinate against COVID-19: results of a nationwide survey. Vaccine.

[21] Reiter PL, Pennell ML, Katz ML (2020). Acceptability of a COVID-19 vaccine among adults in the United States: how many people would get vaccinated?. Vaccine.

[22] Seale H, Heywood AE, Leask J, Sheel M, Durrheim DN, Bolsewicz K (2021). Examining Australian public perceptions and behaviors towards a future COVID-19 vaccine. BMC Infect Dis.

[23] Yoda T, Katsuyama H (2021). Willingness to receive COVID-19 vaccination in Japan. Vaccines (Basel).

[24] Lin Y, Hu Z, Zhao Q, Alias H, Danaee M, Wong LP (2020). Understanding COVID-19 vaccine demand and hesitancy: a nationwide online survey in China. PLoS Negl Trop Dis.

[25] Freeman D, Loe BS, Chadwick A, Vaccari C, Waite F, Rosebrock L (2020). COVID-19 vaccine hesitancy in the UK: the Oxford coronavirus explanations, attitudes, and narratives survey (Oceans) II. Psychol Med.

[26] Sherman SM, Smith LE, Sim J, Amlôt R, Cutts M, Dasch H (2021). COVID-19 vaccination intention in the UK: results from the COVID-19 vaccination acceptability study (CoVAccS), a nationally representative cross-sectional survey. Hum Vaccin Immunother.

[27] Fisher KA, Bloomstone SJ, Walder J, Crawford S, Fouayzi H, Mazor KM (2020). Attitudes toward a potential SARS-CoV-2 vaccine: a survey of U.S. adults: a survey of U.S. adults. Ann Intern Med.

[28] Kourlaba G, Kourkouni E, Maistreli S, Tsopela C-G, Molocha N-M, Triantafyllou C (2021). Willingness of Greek general population to get a COVID-19 vaccine. Glob Health Res Policy.

[29] Wang K, Wong EL-Y, Ho K-F, Cheung AW-L, Yau PS-Y, Dong D (2021). Change of willingness to accept COVID-19 vaccine and reasons of vaccine hesitancy of working people at different waves of local epidemic in Hong Kong, China: repeated cross-sectional surveys. Vaccines (Basel).

[30] COVID-19 vaccination. Cdc.gov. 2021. https://www.cdc.gov/vaccines/COVID-19/index.html. Accessed 9 June 2021.

[31] Coronavirus disease (COVID-19): vaccines. Who.int. https://www.who.int/news-room/q-a-detail/coronavirus-disease-(covid-19)-vaccines?adgroupsurvey=%7badgroupsurvey%7d&gclid=CjwKCAiAm-2BBhANEiwAe7eyFPRIisbgYcsJwL_bi3tz6dG1mT7o1c8SSQ_NJ3rTurM6UpEhEB038hoCAWMQAvD_BwE. Accessed 21 May 2021.

[32] Assessment of Knowledge and Determinants of COVID-19 Vaccines Acceptance in the Lebanese Population. Forms.google. https://forms.gle/fTTsePe9sz9cq82p7. Accessed 9 June 2021.

[33] Paul E, Steptoe A, Fancourt D (2021). Attitudes towards vaccines and intention to vaccinate against COVID-19: Implications for public health communications. Lancet Reg Health Eur.

[34] Islam MS, Siddique AB, Akter R, Tasnim R, Sujan MSH, Ward PR, et al. Knowledge, attitudes and perceptions towards COVID-19 vaccinations: a cross-sectional community survey in Bangladesh. bioRxiv. 2021. 10.1101/2021.02.16.21251802. Accessed 9 June 2021. 10.1186/s12889-021-11880-9 PMC8513387 34645399

[35] lbogle. Understanding patients’ feelings on COVID-19 vaccinations and addressing vaccine hesitancy. Air.org. 2021. https://www.air.org/resource/understanding-patients-feelings-covid-19-vaccinations-and-addressing-vaccine-hesitancy. Accessed 9 June 2021.

[36] Sallam M (2021). COVID-19 vaccine hesitancy worldwide: a concise systematic review of vaccine acceptance rates. Vaccines (Basel).

[37] Urrunaga-Pastor D, Bendezu-Quispe G, Herrera-Añazco P, Uyen-Cateriano A, Toro-Huamanchumo CJ, Rodriguez-Morales AJ (2021). Cross-sectional analysis of COVID-19 vaccine intention, perceptions and hesitancy across Latin America and the Caribbean. Travel Med Infect Dis.

[38] Reno C, Maietti E, Fantini MP, Savoia E, Manzoli L, Montalti M (2021). Enhancing COVID-19 vaccines acceptance: results from a survey on vaccine hesitancy in northern Italy. Vaccines (Basel).

[39] Karlsson LC, Soveri A, Lewandowsky S, Karlsson L, Karlsson H, Nolvi S (2021). Fearing the disease or the vaccine: the case of COVID-19. Pers Individ Dif.

