# Supplementary material for: Assessment of COVID-19 vaccines acceptance in the Lebanese population: a national cross-sectional study
Source: J Pharm Policy Pract. 2022 Jan 11;15:5. doi: 10.1186/s40545-021-00403-x (PMC8749113; doi:10.1186/s40545-021-00403-x)
Supplement: Supplementary file 1 — Additional file 1: Appendix 1. Questionnaire—English Version. [file 40545_2021_403_MOESM1_ESM.docx]

**APPENDIX 1: Questionnaire – English Version**

Dear Participants,

We would like to invite you to take part in our 10-minutes survey about the knowledge and determinants of COVID-19 vaccine acceptance in the general population in Lebanon. Your participation in this research is entirely voluntary. The collected information is anonymous, will be kept confidential, and used for scientific purposes only. We thank you in advance for your valuable help.

Do you want to participate in this study? Yes No

Are you 18 years and older? Yes No

Are you currently living in Lebanon? Yes No

**SECTION I: SOCIO-DEMOGRAPHICS AND MEDICAL HISTORY**

| 1. Gender | Male Female |
| --- | --- |
| 1. 14. Are you pregnant? | No  Yes |
| 1. Are you breastfeeding? | No  Yes ___________________ |
| 1. What is your age (in years)? | ________________ |
| 1. What is your weight? (in Kg)? |  |
| 1. What is your height (in cm)? |  |
| 1. What is your nationality? | Lebanese Syrian Palestian Other: Specify ______ |
| 1. Residence area | Beirut Mount Lebanon North South Bekaa Akkar Nabatieh Baalbak Hermel |
| 1. You are currently living in an urban or rural environment? | Close or around a major city  In a village |
| 1. Marital Status | Single Married Divorced Widowed ______ |
| 1. Living with (check all that applies). | Children  Individuals older than 65 years  Individuals with chronic diseases  It doesn’t apply |
| 1. Education | School level  University level  High degree level (master and doctorate) |

| 1. Occupation | Unemployed  High-risk job: Job with a very high potential for exposure to known or suspected COVID-19 cases (ex: healthcare…)  Medium risk job: Job that requires frequent sustained close contact with other people (supermarkets, pharmacies…)  General risk job: a job that does not require close contact with other people (ex: remote worker, office workers…) |
| --- | --- |
| 1. Are you an active smoker | No  Yes, Smoking cigarettes  Yes, Smoking waterpipe  Yes, Smoking Cigar  Yes, Smoking an e-cigarette |
| 1. Do you have any of the below medical conditions   (More than 1 answer if applicable) | Diabetes  Hypertension  Cardiac Conditions  Thromboembolic disease  Malignancies / Cancers  Respiratory problems such as asthma, Chronic Obstructive Pulmonary Disease (COPD)  Immunodeficiency or Autoimmune disease (ex: rheumatoid arthritis, lupus...)  Kidney or liver disease |
| 1. Do you have any allergies to medications? | No  Yes |
| 1. Do you have any other allergies (food or other)? | No  Yes _______ |
| 1. Have you had, or are you going to have the influenza vaccine? | No  Yes |

**SECTION II: EXPERIENCE WITH COVID-19 INFECTION**

| 1. Have you ever had CO VID-19 infection | No  Yes |
| --- | --- |
| 1. If yes to Q19, kindly specify the severity of symptoms | Mild  Moderate  Severe  It does not apply |
| 1. If yes to Q19, do you suffer from residual symptoms? | No  Yes  It does not apply |
| 1. how much are you scared of getting infected or reinfected by COVID-19 On a scale of 0 to 10? 10 being extremely scared | ________ |
| 1. Do you know any friend or family member who has been hospitalized or have died from a COVID-19 infection | No  Yes |

**SECTION III: KNOWLEDGE**

| Please identify whether the below statements are True or False | True | False | Don’t know |
| --- | --- | --- | --- |
| 1. COVID-19 vaccines decrease the risk of symptomatic infection with the COVID-19 virus |  |  |  |
| 1. COVID-19 vaccines decrease the risk of transmission of the COVID-19 virus |  |  |  |
| 1. All available vaccines produce antibodies against COVID-19 |  |  |  |
| 1. COVID-19 vaccines provide you with immediate protection directly after the first dose |  |  |  |
| 1. Johnson and Johnson's vaccine is given in 2 doses |  |  |  |
| 1. All COVID-19 vaccine preparation techniques are new and have never been used before |  |  |  |
| 1. COVID-19 vaccine is an effective treatment of active COVID-19 infection |  |  |  |
| 1. Most of the confirmed side effects of the COVID-19 vaccines are mild, resolving in 2-3 days |  |  |  |
| 1. People who were previously infected with Covid-19 will need to be vaccinated for COVID-19 at a certain time |  |  |  |
| 1. The faster the people will become vaccinated, the probability of appearance of new variants of the virus will decrease (UK variant, South African, etc…) |  |  |  |
| 1. It is preferable that the two doses of the vaccine given to an individual be from the same brand |  |  |  |
| 1. Vaccinated people will not need to take preventive measures |  |  |  |
| 1. Anyone can take the COVID-19 vaccine |  |  |  |
| 1. Influenza vaccine protects against COVID-19 |  |  |  |
| 1. COVID-19 vaccines contain microchips influencing our body and brain |  |  |  |

1. Where do you get the information about COVID-19 Vaccines?

- Ministry of Public Health website
- World Health Organization (WHO)
- Primary care physician
- Scientists/Scientific releases
- Pharmacists
- Social Platform (Facebook, WhatsApp., etc..)
- Friends/family members
- Television/media website

**SECTION IV: PRACTICE AND BELIEFS**

1. What is your current practice with medicines in general?

- Usually, I take when needed
- Only when prescribed by doctors
- I do not like to take medicine at all

1. Do you oppose vaccination as a concept altogether?

Yes No I don’t know

1. Are you willing to take the COVID-19 vaccine?

I already received at least 1 dose of the vaccine Yes No I didn’t decide

1. Please list why you want to take it, or you have taken it already? (check all that applies)

- Because it may become mandatory for traveling abroad
- Because I want the pandemic to end quickly
- Because I want to protect myself from getting infected with COVID-19
- Because I want to protect my family from getting infected with COVID-19
- Because I am afraid of the severity and complications of COVID-19
- Because I want to return to my normal familial and social life
- Because I want to resume my normal professional activities
- Because I am part of the high-risk group
- Because I want that my children resume school as soon as possible
- Because it is recommended by physicians
- Because it is recommended by the Lebanese Ministry of Public Health
- Because it is recommended by the World Health Organization (WHO)
- Because I believe that the benefits of the vaccination outweigh its possible side effects
- Other reason: (specify)

1. Since you don't want to or are still hesitating about taking the vaccine Please, specify your reasons? (check all that applies)

- I believe that the vaccine may not be effective
- I believe that the vaccine may not be effective with the new strains
- I am concerned about the potential long-term serious side effects of the vaccine
- I am concerned about the short-term side effects COVID-19 vaccines may cause including allergic reactions
- I am concerned about the effects of new technologies (mRNA) used in the vaccine production
- I don’t need to be vaccinated because I am young
- I don’t need to be vaccinated because I am healthy
- I don’t need to be vaccinated because I already got infected by the COVID-19 and this gave me enough immunity
- I don’t need to be vaccinated because I take all the necessary preventive measures
- I don’t want to get vaccinated because I believe that the time allocated for vaccines’ clinical trials was too short
- I don’t want to get vaccinated because I believe that the COVID-19 vaccination is a conspiracy (5G, “micro-chip” implantation, …)
- I don’t want to get vaccinated because I am concerned about the ability of the Lebanese Government to maintain the quality of the vaccine
- I don’t want to take the vaccine because of religious reasons
- Other reasons: specify…

1. Did you register yourself on the MOPH vaccination platform?

Yes No

1. Did you register your family members and/or parents on the platform?

Yes No Not Applicable

1. If you decided not to take the vaccine or you are still hesitant, what do you need to know to make accept the COVID-19 vaccine? (more than one answer can apply)

- I would like to get more information about it
- I would wait to see objective results around me
- If it becomes available in convenient locations outside the hospitals
- If I’m allowed to choose the brand of the vaccine
- If an opinion or religious leaders were vaccinated
- other, specify:

Comment Section: if you have any comments, please share them with us.

_____________________________________________________________________________
